# A Food Box Intervention to Reduce Blood Pressure in Native American Adults With Hypertension: The CHEERS Study

**DOI:** 10.1093/geroni/igab046.2375

**Published:** 2021-12-17

**Authors:** Tori Taniguchi, Joy Standridge, Tyra Shackleford, Hilary Brookins, Tvli Jacob, Valarie Blue Bird Jernigan

**Affiliations:** 1 Center for Indigenous Health Research and Policy at Oklahoma State University Center for Health Sciences; 2 Chickasaw Nation, Ada, Oklahoma, United States; 3 Oklahoma State University Center for Health Sciences, Tulsa, Oklahoma, United States

## Abstract

Diet-related chronic diseases, such as hypertension and obesity, are prevalent in Native American (NA) communities where poor food environments are prominent and healthy food access is limited. The Chickasaw Healthy Eating Environments Research Study (CHEERS) is an NIH-funded study aimed to improve Body Mass Index and blood pressure control among NA adults with uncontrolled hypertension. This multi-level randomized trial, guided by a community-based participatory research orientation, was co-created by tribal and university partners and is implemented within the Chickasaw Nation of Oklahoma. We created hypertension-specific food boxes that contained DASH diet foods, coupons for purchasing vegetables and fruits, educational materials, and heart-healthy recipes for supporting healthy eating. Food boxes were packed and shipped monthly to intervention participants with a 30-day supply of: one fruit serving/day, one vegetable serving/day, one serving of unsalted nuts or seeds/day, one serving of beans or lentils/day, and two servings of fatty fish/week. We will present our participatory approach in co-developing the CHEERS study methods, findings with a focus on older adults, and lessons learned. CHEERS is the first innovative food box intervention to be conducted in NA communities. Food box interventions show promise in improving dietary intake and reducing hypertension and obesity in rural and poor food environments.

